# TB/HIV risk factors identified from a General Household Survey of South Africa in 2006

**DOI:** 10.1080/17290376.2014.912588

**Published:** 2014-05-13

**Authors:** Sathiya Susuman Appunni, Renette Blignaut, Siaka Lougue

**Affiliations:** ^a^ MA, MPhil (Population Studies), PhD (Demography) is Associate Professor in Demography at the Department of Statistics and Population Studies, University of the Western Cape, Cape Town, South Africa; ^b^ PhD (Statistics) is Associate Professor in Statistics and Deputy Chairperson at the Department of Statistics and Population Studies, University of the Western Cape, Cape Town, South Africa; ^c^ PhD (Demography) is Lecturer at the Department of Statistics and Population Studies, University of the Western Cape, Cape Town, South Africa

**Keywords:** socioeconomic, demography, illness/injuries, living spouses, TB/HIV, maladies, VIH/SIDA, tuberculose, socio-économiques, démographiques

## Abstract

The level of human immunodeficiency virus (HIV), tuberculosis (TB) as well as the co-infection TB/HIV in South Africa is among the highest in the world. TB is curable while HIV is not, yet the combination of both is a growing feature in the world. This study examined TB and HIV affecting people living in South Africa. Analyses have been undertaken based on data from the General Household Survey of South Africa in 2006. The study focused on respondents aged 15–49 years, corresponding to a total of 55,384 people composed of 25,859 males and 29,525 females. Among this population, 5935 people suffered from illness/injury, including 2469 (41.6%) males and 3466 (58.4%) females. Weighted multivariate logistic regression is performed on TB and/or HIV in association with the province, background characteristics of the target population, and selected socioeconomic and demographic variables included in the survey. In this study we focus on variables of health status and whether subjects suffered from TB and/or HIV. Findings of this investigation show that TB is the second most common cause of illness in the provinces of KwaZulu-Natal (KN) (9.1%), North West (5.4%) and Limpopo (4.2%). People who are married have a 50% lower risk compared to those currently not married to suffer from TB and/or HIV. Those with living spouses have a 5% lower risk to suffer from TB and/or HIV than those whose partners are not alive. This study concluded that rapid action is needed to curb the spread of TB and/or HIV to produce a healthy population. Therefore, follow-up care and special preventative measures are urgently needed in provinces with higher reported rates of TB and/or HIV such as KN.

## Résumé

Cette étude examine les maladies affectant la population de l'Afrique du Sud. L'objectif de l’étude est d'examiner les facteurs à risque de TB/VIH à partir d'une enquête générale auprès des ménages de l'Afrique du Sud en 2006. Plus précisément, nous mettons l'accent sur les variables de l’état de santé et si les sujets ont souffert de la tuberculose ou du VIH. L’étude a porté sur les répondants âgés de 15–49 ans, correspondant à un total de 55 384 personnes, composée de 25 859 hommes et 29 525 femmes . Parmi cette population, 5935 personnes ont souffert de maladie/blessure avec 2469 (41,6%) des hommes et 3466 (58,4%) femmes. Cet article fournit des estimations multivariées pondérés de la tuberculose (TB) et/ou le virus de l'immunodéficience humaine (VIH) par province en association avec les caractéristiques de base de la population cible et certaines variables socio-économiques et démographiques. La tuberculose est la deuxième cause de maladie dans les provinces du KwaZulu Natal (9,1%), du Nord-Ouest (5,4%) et le Limpopo (4,2%) . Les personnes mariées ont 50% moins de risque de souffrir de la tuberculose et/ou du VIH que les mariées . En outre, ceux qui ont leur conjoint encore en vie ont 5% moins de risque de souffrir de la tuberculose et/ou du VIH que les veuves et veufs. En général, cette étude a montré que les personnes ayant un conjoint ou un parent vivant ont un risque plus faible de maladie de la tuberculose/VIH par rapport à ceux qui ne sont pas mariés ou ne n'ont pas de parents en vie. Les conclusions de cette étude militent pour une action rapide est nécessaire pour enrayer la propagation de la tuberculose et du VIH afin de produire une population saine. Par conséquent, les suivi des soins et des mesures spéciales de prévention sont nécessaires d'urgence dans les provinces de haute prevalence de tuberculose et/ou VIH signalés comme le KwaZulu Natal.

## Introduction

According to the General Household Survey (GHS) of South Africa (2006), illness and injury have been measured using the following categories: flu or acute respiratory tract infection, diarrhea, severe trauma, tuberculosis (TB) or severe cough with blood, abuse of alcohol or drugs, depression or mental illness, diabetes, high or low blood pressure, human immunodeficiency virus (HIV)/AIDS, other sexually transmitted diseases (STDs) and all other illnesses and injuries are grouped into a final category referred to as ‘Other illnesses and injuries’. Some diseases develop fast after infection but also end speedily such as cold or flu. Other illnesses, such as asthma or diabetes, are chronic, which means that they last a long time, perhaps even a lifetime. Although this article will report on illness and injury occurrence per province, the main section of this article investigates factors related to TB and/or HIV. South Africa faces a quadruple burden of maladies consisting of HIV and AIDS, communicable diseases, non-communicable diseases and violence and injuries. The consequence of this is high levels of mortality and morbidity (Motsoaledi 2010). In 2009, Statistics South Africa estimated the life expectancy of South Africans to be 54 years for males and 57 years for females.

South Africa has the third highest TB burden in the world (World Health Organization 2010, 2012). TB is a curable disease which is emerging due to HIV/AIDS. The co-infection of TB and HIV has been reported by many studies as a major concern (Shisana, Labadarios, Rehle, Simbayi, Zuma, Dhansay, *et al*. 2013). Regarding the burden of TB and/or HIV in South Africa, this study was initiated to improve the knowledge about TB/HIV by focusing on the available information collected by the GHS of South Africa (2006). This study describes factors that are associated with HIV and/or TB from an epidemiological perspective in South Africa. Factors included in the model are more prevention related. Prevention is better than cure, but sometimes disease prevention is the only available alternative.

The prevalence of HIV varies by wealth status, education attainment, occupation and type of residence (rural /urban); for example, 61% of adults living with HIV in sub-Saharan Africa in 2007 were women (Faber 2007). Another important subject is food availability, which was identified as the most immediate and critical need for people living with HIV/AIDS (Steyn, Bradshaw, Norman, Joubert, Schneider, & Steyn 2006). Malnutrition itself can induce immune-depression and worsen the conditions of people living with HIV. In South Africa, an epidemiological transition is taking place with a shift in disease burden from infectious diseases such as HIV and/or TB to chronic diseases (Health Opportunities for People Everywhere 2011).

The latest report of World Health Organization (2012) also mentioned that in South Africa, non-communicable diseases and disorders related to a person's way of life, such as diabetes, stroke and cancer, are rapidly gaining momentum as the cause of illness. The main problem was people living in low status of life, malnutrition, anemia, famine, hunger, poverty also increases the risk of vulnerable diseases through vitamin A and iodine deficiencies, poor nutrition, bad working conditions, poor sanitation, environmental pollution and lack of health care (Sen & Wolfensohn 2005) and researchers from diverse professions such as demographers, social scientists, medical scientists, human rights officials, etc., are displaying keen interest in the subject of illness (Desai 1990). Unfortunately, data on morbidity in South Africa are inadequate (General Household Survey 2006; Kapteyan 2004; Population Policy 1998; South Africa Year Book 2007/2008; World Health Organization 1980, 1985). This study reports on a quantitative-based analysis using weighted data to investigate illness/injuries, with particular reference to TB and/or HIV in the age group of 15–49 years, in all the provinces of South Africa. In addition, findings of the study will contribute to improving population health and provide useful information for planners and policy-makers.

## Data and methods

### Data

The South Africa GHS (2006) is the large-scale data source used in this article. The survey selected a sample as per the multi-stage stratified sampling method. The sample size was composed of 3000 primary sampling units (PSUs) allocated to the 53 district councils using the power allocation method. PSUs are enumeration areas (EAs) from the census list that had a household count of more than 25 excluding workers' hostels, convents and monasteries. In addition, EAs in the census that have less than 60 dwelling units were combined to form PSUs.

The PSUs were sampled in each district using a probability proportional to the number of households in a PSU as calculated in the census. In each PSU, dwelling units were selected using a systematic sampling technique (General Household Survey 2006). The GHS is a survey conducted in South Africa with three major data sets: household, persons and workers files. There are nine provinces in South Africa: Western Cape (WC), Eastern Cape (EC), Northern Cape (NC), Free State (FS), KwaZulu-Natal (KN), North West (NW), Gauteng (Gau), Mpumalanga (Mpu) and Limpopo (Lim). The interviews concerned 32,146 households and 87.5% of the interviews were successfully completed.

Because HIV is an STD and the co-infection TB/HIV is of particular interest in this study, the population under study comprised respondents of age group 15–49 years, corresponding to an adult population in a sexually active age group. This population is composed of a total of 55,384 people, including 25,859 males and 29,525 females. Among this population, 5935 people suffer from illness/injury including HIV/TB where 2469 (41.6%) were males and 3466 (58.4%) were females.

### Methods

This study performed an advanced statistical method of the General Household Survey data. The analyses consisted of three different weighted multivariate analyses corresponding to the three dependent variables TB, HIV and TB/HIV. Since all the dependent variables are dichotomous (yes, no), logistic regression was used as the appropriate statistical technique in such circumstances. The dependent variable was whether a person indicated that he/she suffered from either HIV and/or TB. A stepwise logistic regression procedure for each dependent variable in association with background characteristics of the target population, and selected socioeconomic and demographic variables were undertaken. Independent variables included in the stepwise logistic regression procedure were as follows: province, gender, marital status, highest educational level, father alive, mother alive, illness/injuries, whether subject consulted a health worker, kind of health worker consulted, type of health institution and disabilities present or not.

## Results

The distribution of illness/injuries according to the type of illness/injuries and province highlighted many important facts and crucial aspects that deserve special focus and in-depth analysis. Findings from Table 1 show that flu or acute respiratory tract infection is the major cause of illness/injuries in the country and accounts for about half of the reported illness in the month prior to the survey (48.9% and 51.9% for unweighted and weighted, respectively). The most fascinating and concerning result is that the second place is occupied by TB, with 6.7% for unweighted results and 5.2% when weighted.

**Table 1.** Percentage distribution of respondents in the age group of 15–49 who suffered illness/injuries in South Africa by provinces.

Provinces	1	2	3	4	5	6	7	8	9	10	11
*Unweighted*											
WC	40.8	2.9	4.0	8.4	0.4	2.7	3.5	7.5	1.6	0.2	27.9
EC	38.9	7.6	4.5	7.8	1.0	4.6	3.2	7.5	2.7	0.8	21.5
NC	44.3	2.2	3.5	7.0	0.5	4.1	3.5	8.9	2.7	1.4	21.9
FS	49.9	1.3	2.4	5.0	0.4	4.0	1.9	8.0	2.4	0.4	24.3
KN	46.6	4.5	3.6	10.6	0.8	4.5	4.2	4.2	2.2	0.8	18.1
NW	45.9	1.8	3.2	6.9	0.6	5.9	3.3	4.7	2.3	0.3	25.1
Gau	61.7	1.9	3.2	3.0	0.5	1.8	2.0	5.2	1.4	0.0	19.2
Mpu	55.6	3.2	3.1	4.3	0.1	2.8	2.8	4.0	1.1	0.7	22.4
Lim	56.1	2.0	2.5	3.9	0.0	4.3	1.6	3.6	0.2	0.9	24.8
Total	48.9	3.4	3.4	6.7	0.6	3.9	3.0	5.7	1.9	0.6	21.9
*Weighted*											
WC	41.2	3.3	2.7	6.1	0.2	8.1	3.8	7.1	0.8	0.1	26.6
EC	38.7	8.6	5.4	7.3	1.2	3.9	3.0	6.6	2.5	0.7	22.2
NC	42.9	2.2	2.4	6.8	0.5	4.0	3.4	7.6	3.1	1.2	26.1
FS	55.5	1.4	2.9	3.3	0.4	3.4	1.4	6.7	2.0	0.4	22.7
KN	50.2	3.5	4.1	9.1	0.7	4.5	4.2	4.0	2.0	0.9	16.8
NW	44.1	1.5	4.5	5.4	0.3	5.1	2.4	3.8	2.3	0.6	30.0
Gau	63.4	1.8	4.5	2.6	0.3	1.6	1.7	4.1	0.8	0.0	19.2
Mpu	56.2	3.3	3.0	3.6	0.2	2.4	2.7	4.3	1.0	0.5	22.9
Lim	57.6	2.1	1.4	4.2	0.0	4.1	1.9	3.9	0.2	0.8	23.8
Total	51.9	3.3	3.9	5.2	0.5	3.7	2.6	5.0	1.5	0.5	22.0

Source: General Household Survey (2006).

Notes: 1. Flu or acute respiratory tract infection, 2. diarrhea, 3. severe trauma, 4. TB or severe cough with blood, 5. abuse of alcohol or drugs, 6. depression or mental illness, 7. diabetes, 8. high or low blood pressure, 9. HIV/AIDS, 10. other STD and 11. other illness or injury.

The province of KN reported the highest percentage of illness due to TB with 9.1%, followed by the provinces of EC (7.3%) and the NC (6.8%). The provinces of Mpu, FS and Gau recorded the lowest percentages of TB among people living with illnesses/injuries, at 3.6%, 3.3%, and 2.6%, respectively. However, it is important to mention that the percentage of reported TB is low in Lim (4.2%), but it is the third highest cause of illness/injuries after flu or acute respiratory tract infection. However, in provinces such as WC, EC, and NC, the percentage of reported TB is the fourth or fifth most common cause of illness. In the WC, for instance, with 6.1%, TB represents the fifth most common cause of illness/injuries after flu symptoms (41.2%), unspecified illnesses (26.6%), depression or mental illness (8.1%) and high or low blood pressure (7.1%). In brief, TB is the third most common cause of illness/injuries in the provinces of KN (9.1%), NW (5.4%) and Lim (4.2%). In the WC, depression or mental illness, at 8.1%, is the third greatest cause of illness/injuries, while in the EC it is diarrhea with 8.6%. In the provinces of NC, FS and Mpu, the third position is occupied by high or low blood pressure, with 7.6%, 6.7% and 4.3% of illness/injuries, respectively. In Gau severe trauma (4.5%) was reported as the third highest cause of illness/injuries. Findings from this study highlight TB and HIV as important problems in South Africa. In Table 2, the results of three models are shown: in Model I reported TB is defined as the dependent variable, in Model II, reported HIV is defined as the dependent variable and in Model III, TB and /or HIV is defined as the dependent variable. Table 2 presents the independent variables included in the stepwise logistic regression procedure.

**Table 2.** Logistics regression of 15–49 years who suffered TB, HIV and TB/HIV with background variables in South Africa.

	Model I (TB)			Model II (HIV)			Model III (TB/HIV)		
	Exp(B)	95% CI for Exp(B)		Exp(B)	95% CI for Exp(B)		Exp(B)	95% CI for Exp(B)	
		Lower	Upper		Lower	Upper		Lower	Upper
Sex									
Female^a^									
Male	0.978***	0.968	0.988	0.312***	0.305	0.319	0.804***	0.797	0.812
Marital status									
Others^a^									
Currently married	0.525***	0.516	0.534	0.424***	0.410	0.438	0.501***	0.493	0.508
Spouse alive									
No^a^									
Yes	0.988***	0.974	1.002	0.796***	0.774	0.818	0.953***	0.941	0.965
Father alive									
No^a^									
Yes	0.453***	0.448	0.458	0.557***	0.545	0.568	0.475***	0.470	0.479
Mother alive									
No^a^									
Yes	0.622***	0.615	0.629	0.463***	0.454	0.472	0.592***	0.586	0.598
Disabilities									
No^a^									
Yes	3.490***	3.432	3.548	8.988***	8.784	9.196	4.338***	4.278	4.398
Constant	0.013***			0.006***			0.018***		

Source: General Household Survey (2006).

^a^Reference category.

*Level of significance at *P* < .05.

**Level of significance at *P* < .01.

***Level of significance at *P* < .001.

All models reported very similar results. All selected background variables are significant at a 1% level of significance for all the models. Findings show that males are at lower risk compared to females to contract TB and or HIV. Married people seem to be less likely compared to unmarried people to get TB and or HIV. Table 2 revealed that having a mother or father alive decreases the risk of having HIV and or TB. Categories including live fathers and live mothers show more or less similar results, with 53% and 41% lower risk for people with mothers or fathers alive than the reference category. People living with disability have a 3.5 times higher risk contracting TB and 9 times higher risking contracting HIV. Those with living spouses have a 5% lower risk to suffer from TB and/or HIV than those who do not have a partner or their partner is not alive.

## Discussion

Findings of this article confirm the high level of association between province of residence and risk of HIV/TB. The significance of the influence has been established. The main finding of this study is that the influences of risk factors on HIV and or TB are direct and also indirect via other background factors such as age, sex, living or dead partner, marital status, number of living children, children ever born, and ability to read and write.

The increased number of people suffering from illness illustrates the important needs in this domain because these people have problems that are different from others. The number of people living with illness is quite high in all the provinces, but more people in KN province are suffering from HIV/AIDS, TB (or severe cough with blood 9.2%) and other illnesses of South Africa. Biases and insight due to the lack of knowledge and prevention about TB/HIV among particular population such as people living with a disability are shown to be lacking. Many reviews of literature show that most of the people in the age group 15–49 years suffer from TB and or HIV due to lack knowledge about the prevention methods, symptoms and treatment (Shisana *et al.* 2013). Some findings of this study are very important and should be understood in their context. The lower likelihood of contracting TB/HIV among married people and those who have their spouse alive highlights the reality that most of those having HIV/TB already lost their partner due to the disease. Marriage is also appearing as a means of reducing the risk of HIV and or TB. In general, married people and those who have their parents alive are less at risk of TB/HIV because they have someone close enough to help them to follow the treatment of TB and or HIV. The access to health services is also important for TB treatment and cure. Regarding HIV it can be considered as a proxy of access to knowledge, prevention and treatment as well. The goal of improving health outcomes and accelerating progress toward MDGs is not solely dependent on health sector interventions, but is also determined by factors that lie outside the health sector, such as access to education, water and sanitation, among others. Concerted actions between health, education and other sectors are required to accelerate progress toward achievement of the health-related MDGs.

## Conclusions

The relationship between TB/HIV and respondents' socioeconomic and demographic variables is significant and respondents' wealth index and key demographic factors play an important role. The relationship between TB/HIV and the hidden possible factors has not been sufficiently explored by scientists. Toward the goal of contributing to fill this gap, this article has been initiated to examine the risk factors of HIV and or TB with an emphasis on the sense and the importance of the influence they have on each other. The purpose of this work was to contribute to the understanding of TB/HIV in South Africa in order to change the image of people living with it in the society. The multivariate analysis showed that it is possible to reduce TB and or HIV through actions targeting high-risk population without neglecting the others and also focusing on knowledge spreading. Rapid action is needed to fight against illness/diseases to give our country a healthy population. More financial support is needed in provinces such as KN that are weaker in terms of sickness or illness. Steps need to be taken to expand basic amenities such as transportation and primary health centers through proper medical preventive services. Ensure that poor rural people are able to receive appropriate treatment or gain access to primary health services in the case of any emergencies. This study led to results that should be further explored in future studies for a better understanding of TB/HIV. It would be interesting to initiate an in-depth investigation, such as a qualitative-based study on this issue taking into account more respondent background variables. These are among other factors that could allow us a clear-cut understanding and achieve more visibility for the issue of illness.
